# Visual impairment following aluminum phosphide poisoning: A rare case

**DOI:** 10.1002/ccr3.7422

**Published:** 2023-06-07

**Authors:** Alireza Kargar, Maryam Noroozian, Mahtab Ramezani, Shahin Shadnia, Babak Mostafazadeh, Peyman Erfan Talab Evini, Mitra Rahimi

**Affiliations:** ^1^ Cognitive Neurology and Neuropsychiatry Research Center Tehran University of Medical Sciences (TUMS) Tehran Iran; ^2^ Department of Clinical Pharmacy School of Pharmacy Shahid Beheshti University of Medical Sciences (SBMU) Tehran Iran; ^3^ Cognitive Neurology and Neuropsychiatry Division Department of Psychiatry Tehran University of Medical Sciences (TUMS) Tehran Iran; ^4^ Department of Neurology, School of Medicine Tehran University of Medical Sciences (TUMS) Tehran Iran; ^5^ Department of Clinical Toxicology Toxicological Research Center Excellence Center of Clinical Toxicology Loghman Hakim Hospital Shahid Beheshti University of Medical Sciences (SBMU) Tehran Iran

**Keywords:** aluminum phosphide, cardiogenic shock, cerebral atrophy, hypoperfusion, poisoning, visual loss

## Abstract

**Key Clinical Message:**

Aluminum phosphide poisoning may cause rare visual impairment. In a case, a 31‐year‐old female, visual loss was linked to shock‐induced hypoperfusion, causing oxygen lack and cerebral atrophy, emphasizing the need for identifying atypical symptoms.

**Abstract:**

This case report describes the multidisciplinary evaluation of a 31‐year‐old female patient who suffered from visual impairment as a result of aluminum phosphide (AlP) poisoning. Phosphine, which is formed in the body when AlP reacts with water, cannot cross the blood–brain barrier; therefore, visual impairment seems unlikely to be the direct result of phosphine. To our knowledge, it is the first documented report of such impairment due to AlP.

## Graphical abstract

This case report illustrates a rare consequence of aluminum phosphide (AlP) poisoning ‐ visual impairment ‐ in a 31‐year‐old female. In the pursuit of understanding the underlying pathophysiology, MRI scans were performed. The axial FLAIR sequence (A) revealed bilateral cortical hyperintensity, while the sagittal T1‐weighted sequence (B) showed no signal abnormality. While the toxic compound phosphine released by AlP cannot cross the blood‐brain barrier, the impairment was attributed to cerebral atrophy due to shock‐induced hypoperfusion.

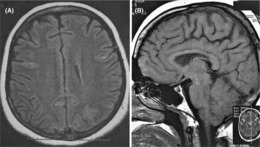

## 1 INTRODUCTION

Aluminum phosphide (AlP) is a widely recognized pesticide, serving as one of the most efficacious toxic compounds in the marketplace for the defense of stored grains against infestations by rodents and diverse pest species. There are a large number of AlP poisoning cases in Iran,^1^ and the mortality rate ranges from 30% to 77% within 1–2 days of admission due to AlP poisoning; as a result, AlP poisoning makes up a large number of poisoning cases in Iran.^1^, ^2^ Suicide is the most common cause of AlP poisoning in Iran.^3^ Several countries in Europe and the United States have registered this product for the fumigation of agricultural compounds, feed for animals, and processed foods, as well as outdoor pest control. In Iran, this product is available in some herbal groceries.^4^ In developing countries, it is popular due to its low cost, convenience, and high effectiveness.^1^ In studies, 150 mg–500 mg of this compound was found to be the lethal dose.^5^

## 2 CASE PRESENTATION

A 31‐year‐old, right‐handed female patient presented to our emergency department following intentionally consuming AlP for a suicide attempt. Six hours after drinking a glass of water containing two dissolved AlP tablets, she developed nausea and abdominal pain.

### 2.1 Clinical manifestations and para‐clinical findings

Upon admission, she was fully conscious (Glasgow Coma Scale 15/15). Examination of vital signs revealed a respiratory rate of 20 per minute (20/min), a heart rate of 122 beats/min, blood pressure of 99/60 mmHg, temperature of 36.8°C, O_2_ saturation of 100%, and blood glucose of 102 mg/dL. Pupils were similar, midsized, nystagmus‐free, and reactive to light. The general physical examination was unremarkable.

Her first venous blood gas (VBG) analysis showed mild metabolic acidosis with pH = 7.23, HCO_3_ = 21 mEq/L, and pCO_2_ = 49.6 mmHg. It was decided to intubate the patient due to metabolic acidosis that occurred as a result of persistent hypotension and supportive treatment was initiated for her. During AlP poisoning, the onset of metabolic acidosis serves as confirmation of intoxication. In anticipation of potential loss of consciousness and subsequent vital organ failure within the following hours, the patient is preemptively intubated to safeguard their physiological stability.

**FIGURE 1:**
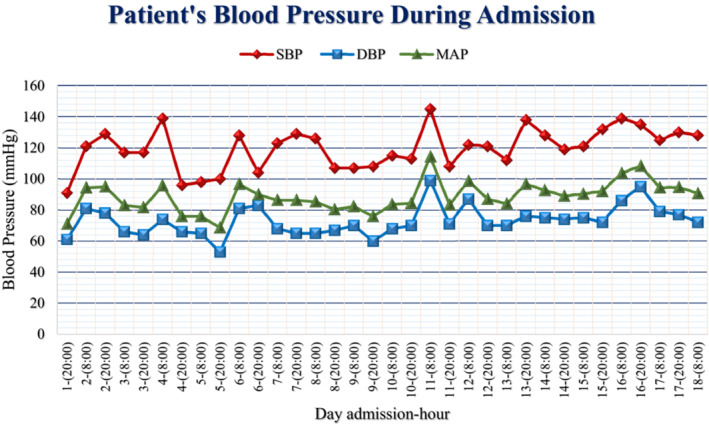
Patient's blood pressure during admission; it is clear from the chart that the patient has experienced many fluctuations in his blood pressure. In comparison with other days of hospitalization, the patient's blood pressure dropped more upon the fourth and fifth days of hospitalization. DBP, diastolic blood pressure; MAP, mean arterial pressure; SBP, systolic blood pressure.

Variability in blood pressure during the admission is shown in Figure 1. Several laboratory tests during hospitalization are shown in Table 1.

**TABLE 1:** Laboratory and clinical data of the patient during her admission.

Admission day	1	2	3	4	5	6	7	8	9	10	11	12	13	14	15	16	17	18
O_2Sat_ (%)	97	98	98	95	94	92	93	96	97	97	92	94	98	96	97	98	98	97
HR (beat/min)	124	136	130	132	95	88	131	130	108	120	107	100	63	70	69	110	86	83
pH	7.176	7.23	7.171	7.198	7.3	7.412	7.347	7.522	7.521	7.462	7.402	7.447	7.484	‐	7.494	7.463	7.485	7.41
pCO_2_ (mmHg)	68.1	49.6	71.5	73	51	53	56	43	40	38.6	49	37.7	39.2	‐	31.3	28.5	26.6	35.5
HCO_3_ (mEq/L)	25.2	21	19.3	26	21	33.8	30.9	34.9	31.6	27.6	30.8	26	28.7	‐	24.1	21.8	22.7	22.7
GCS	15	7	7	7	7	7	7	6	6	7	7	10	9	11	12	13	12	12
Mechanical ventilation		SIMV	SIMV	SIMV	SIMV	SIMV	SIMV	SIMV	SIMV	SIMV	SIMV	SIMV	SIMV	SIMV				

Abbreviations: GCS, Glasgow Coma Scale; HCO_3_, bicarbonate; HR, heart rate; O_2Sat_, oxygen saturation; pCO_2_, partial pressure of carbon dioxide; pH, potential of hydrogen; SIMV, synchronized intermittent mandatory ventilation.

### 2.2 Treatment course

Protocol of high‐dose insulin euglycemic therapy was started for the patient. It included a combination of insulin, glucose, and potassium chloride to enhance insulin's positive inotropic and cardioprotective properties. According to the results of VBG and prediction of the deterioration of the patient's condition and to reduce the pressure on the heart, the patient underwent mechanical ventilation. A supportive package including N‐acetylcysteine, vitamin E, calcium gluconate, and magnesium sulfate was also started for the patient. After 14 days, the patient regained consciousness and was extubated.

In the first 2 days after extubation, she did not speak at all. On the third day after her extubation, she complained to the treatment staff that she had suffered some degree of vision loss and was unable to see clearly. Both of the patient's eyes had 1/10 visual acuity, which means that both of her eyes were severely impaired. An ophthalmological examination and optical coherence tomography revealed that the structures of the eyes were intact. There were no abnormalities in the lens or retina of the eyes. A detailed neurological assessment was carried out which showed no abnormal findings. No interactions bearing clinically significant consequences were detected between the prescribed pharmaceutical treatment for the patient. This particular complication was determined to be unrelated to the administration of the medications during hospitalization. In her medical history, she mentions that she experienced a seizure following the injection of penicillin at the age of three.

**FIGURE 2:**
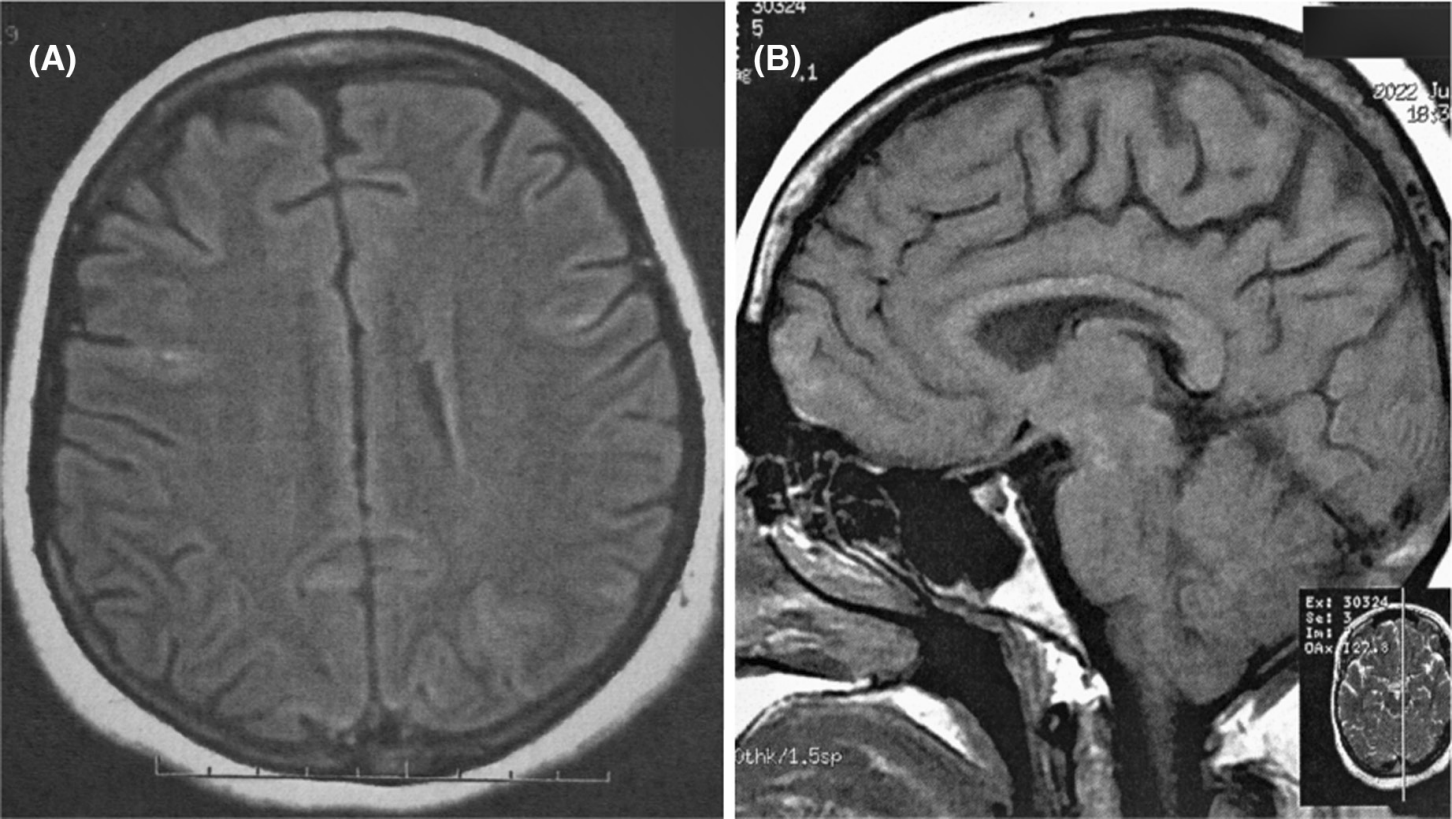
Axial FLAIR sequence reveals bilateral cortical hyperintensity (A), the sagittal T1‐weighted sequence shows no signal abnormality (B).

The patient underwent comprehensive diagnostic evaluations, including a brain and orbital magnetic resonance imaging (MRI) scan and a visually evoked potential (VEP) study, to identify any potential injuries to the optic nerves or eye‐related cortical regions. The orbital MRI, performed with and without gadolinium, and the VEP study did not reveal any pathologic findings. Papillitis and optic neuritis were additionally excluded through the utilization of MRI of the orbital region, as well as the comprehensive ophthalmological assessments conducted at the Farabi Ophthalmology Hospital, a specialized institution in the field. However, the brain MRI demonstrated multiple diffusion restrictions, both cortically and subcortically, in both cerebral hemispheres, which could be associated with prior global hypoperfusion events (Figure 2).

A psychiatrist visited the patient to evaluate her psychological state as part of her routine examination following a suicide attempt involving the ingestion of AlP.

Despite her aspirations to pursue a career in medicine, the patient was unable to attain the necessary score on the entrance examination. Throughout their conversation, the patient exhibited signs of nervousness, indifference, and increased irritability.

Also, in the psychiatric visit, the patient's main complaint was that she had visual impairment due to suicide with AlP. The first time, about 15 years ago, when she was still studying in her high school years, a psychiatrist prescribed her sertraline in a dose of 100 mg. At present, she smokes one pack of cigarettes per day. In the past year, she has been treated with clonazepam and carbamazepine. Additionally, she underwent a rhinoplasty procedure for aesthetic purposes 3 years ago.

The patient reported engaging in frequent verbal disputes with those around her, contributing to an unstable environment. She believes that her working memory and calculation abilities have deteriorated following her suicide attempt and that she now experiences difficulties walking and maintaining balance. The patient was diagnosed with mood disorder type II with atypical major depression based on the report of previous episodes of hypomania. Citalopram and lamotrigine were administered to the patient. Moreover, participation in psychotherapy sessions under the supervision of a psychiatrist was advised.

The patient underwent psychiatric care and psychotherapy as part of a comprehensive treatment plan. Over a one‐year period, she was closely monitored through regular evaluations to ensure her well‐being. At the end of the year‐long follow‐up, the patient exhibited substantial progress in her overall condition.

A subsequent assessment of her eyesight in both eyes indicated a remarkable improvement, as each eye achieved a visual acuity of 9/10. In her most recent consultation, the patient expressed concern about her reduced ability to quickly read messages on her smartphone.

## 3 DISCUSSION

Upon exposure to moisture, AlP tablets release phosphine gas, which is catalyzed by stomach acid. As a result of the absorption of phosphine through the respiratory or gastrointestinal tract, phosphine causes toxicity by inhibiting cytochrome c oxidase and producing highly reactive free radicals.^6^ Consuming AlP results in the release of phosphine gas, which non‐competitively inhibits electron transport chains in cytochrome c oxidase, resulting in diffuse hypoxia of the cells. As of now, there is no antidote for AlP poisoning. Patients are treated with mechanical ventilation and vasopressors, as well as supportive measures.^7^ AlP poisoning rarely results in neurological effects other than headache and confusion.^8^ Herein, we report visual loss as a rare complication of AlP poisoning.

Several symptoms associated with this poisoning are widely observed, such as dizziness, restlessness, anxiety, ataxia, numbness, paresthesia, and tremor. However, central nervous system manifestations are not evident until a secondary event, such as hypoxia, occurs. As a result of late and severe neurologic findings, some rare cases are reported who experienced convulsions, delirium, ischemic/hemorrhagic stroke, and coma. A rare consequence of AlP poisoning can be visual impairment.

Phosphine does not cross the blood–brain barrier.^9^ Consequently, the cortical and subcortical atrophy of the brain is probably not due to the direct effect of phosphine on the brain.

During AlP poisoning, cardiogenic shock can occur, which can explain the occurrence of this complication. Hypoxia after cardiac arrest has been reported to result in visual loss.^10^ Although these cases are very rare, hypoperfusion caused by cardiogenic shock can be considered as one possible explanation for visual impairment in this case. Hypoperfusion can eventually cause cerebral atrophy as a consequence of the patient's oxygen deprivation.

There currently exists no established therapeutic intervention for addressing this issue. It is imperative to diligently endeavor to avert cerebral hypoperfusion throughout the patient's hospitalization period.

## 4 CONCLUSION

In conclusion, AlP poisoning, while typically presenting with common symptoms, can lead to rare neurological complications like visual impairment. This is likely not due to the direct effect of phosphine on the brain, as it does not cross the blood–brain barrier. Instead, cardiogenic shock‐induced hypoperfusion and subsequent oxygen deprivation may eventually result in cerebral atrophy and visual disturbances. While such cases are rare, it is crucial for healthcare professionals to be aware of and address this potential complication when managing patients with AlP poisoning.

## CONSENT

We had obtained written consent in the case.

## AUTHOR CONTRIBUTIONS

**Alireza Kargar:** Writing – original draft; writing – review and editing. **Maryam Noroozian:** Investigation; writing – review and editing. **Mahtab Ramezani:** Writing – review and editing. **Shahin Shadnia:** Supervision. **Babak Mostafazadeh:** Supervision. **Peyman Erfan Talab Evini:** Supervision. **Mitra Rahimi:** Conceptualization; data curation; investigation; writing – review and editing.

## FUNDING INFORMATION

In the interest of full transparency, we would like to clarify that this case report was undertaken and completed without the support of any external or internal funding. All efforts and resources involved in this research were contributed voluntarily by the authors or obtained from already existing resources within our respective institutions.

## Data Availability

All pertinent data for this case study have been made available while maintaining patient confidentiality and anonymity.
